# In Vivo Optofluidic Switch for Controlling Blood Microflow

**DOI:** 10.1002/advs.202001414

**Published:** 2020-06-09

**Authors:** Xiaoshuai Liu, Qing Gao, Yao Zhang, Yuchao Li, Baojun Li

**Affiliations:** ^1^ Institute of Nanophotonics Jinan University Guangzhou 511‐443 China

**Keywords:** blood microflow, optical tweezers, optofluidic manipulation, red blood cells

## Abstract

Control of blood microflow is crucial for the prevention and therapy of blood disorders, such as cardiovascular diseases and their complications. Conventional control strategies generally implant exogenous synthetic materials into blood vessels as labeling markers or actuating sources, which are invasive and incompatible with biological systems. Here, a label‐free, noninvasive, and biocompatible device constructed from natural red blood cells (RBCs) for controlling blood microflow in vivo is reported. The RBCs, optically manipulated, arranged, and rotated using scanning optical tweezers, can function as an optofluidic switch for targeted switching, directional enrichment, dynamic redirecting, and rotary actuation of blood microflow inside zebrafish. The regulation precision of the switch is determined to be at the single‐cell level, and the response time is measured as ≈200 ms using a streamline tracking method. This in vivo optofluidic switch may provide a biofriendly device for exploring blood microenvironments in a noncontact and noninvasive manner.

## Graphical abstract

An optofluidic switch, constructed from natural red blood cells and manipulated by optical tweezers, is proposed to control blood microflow in vivo at the single‐cell level. The flow rate and direction of the blood are determined by controlling local blood pressure using the optofluidic switch, providing a biocompatible tool for exploring blood microenvironments in a noncontact and noninvasive manner.

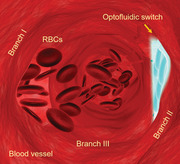

## 1 Introduction

In a healthy person, blood microflow extends optimally into all biological tissues to meet metabolic and homeostatic demands, replenishing fresh nutrients, and removing waste products in vivo.^[^ ^1^ ^]^ Disorders in blood microflow cause serious diseases, including atherosclerosis, thrombus, diabetes, Alzheimer disease, and tumors.^[^ ^2^, ^3^, ^4^, ^5^, ^6^, ^7^, ^8^, ^9^ ^]^ To investigate the underlying mechanism of these blood disorders, considerable interests have been focused on the development of techniques to control blood microflow in vivo, which provide additional insights for clinical diagnosis, drug delivery, and anticancer therapy.^[^ ^10^, ^11^, ^12^ ^]^ Traditional manipulation techniques, which are generally based on magnetic,^[^ ^13^ ^]^ acoustic,^[^ ^14^, ^15^ ^]^ and electrical devices,^[^ ^16^, ^17^ ^]^ have become powerful tools for blood microflow control. However, these techniques require implantation of exogenous materials, such as magnetic nanoparticles, ultrasonic sources or metal electrodes, into the blood to function as labeling markers or actuating sources, and these materials are inevitably incompatible with biological systems. To control blood microflow in a non‐invasive way and with high precision, a biocompatible and single‐cell‐level strategy is highly desirable.^[^ ^18^, ^19^, ^20^ ^]^

Optofluidics, emerging as a fascinating combination of photonics and microfluidic techniques, has seen considerable advancements in the single‐cell‐precision control of cell behavior.^[^ ^21^, ^22^, ^23^, ^24^, ^25^, ^26^, ^27^ ^]^ These control techniques have been further developed as an optofluidic switch to control microfluidic motion.^[^ ^28^, ^29^, ^30^, ^31^, ^32^ ^]^ Aside from artificial microfluidic systems, blood vessels can be regarded as natural microfluidic channels, inside which red blood cells (RBCs) are the most important and abundant components. Using optical tweezers, RBCs can be manipulated in vivo, and arranged into various patterns in a non‐contact and non‐invasive way.^[^ ^33^, ^34^, ^35^, ^36^ ^]^ Moreover, the specific movement and arrangement of RBCs has a major influence on blood microfluidics.^[^ ^37^, ^38^, ^39^ ^]^ By combining the single‐cell precision of optofluidic technique and the non‐invasiveness of optical tweezers, we developed an optofluidic switch using natural RBCs to perform multifunctional control of blood microflow. Unlike traditional control methods, the RBC‐based switch does not require external device implantation, and has no detectable tissue damage, thus providing a biocompatible tool for exploring blood microenvironments in vivo.

## 2 Results

### 2.1 Schematic Illustration and Experimental Setup

**Figure 1:**
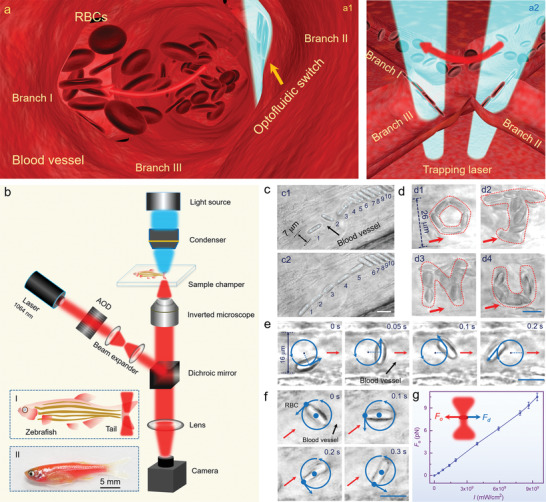
Schematic illustration and characterization of the optofluidic switch. a) Schematic illustration of the assembled optofluidic switch at the entrances of two intersecting blood vessels (a1), and the aerial view for the cross section (a2). b) Schematic illustration of the experiment setup. c) Specific orientation and dynamic shift of ten RBCs in the capillary with a diameter of 7 µm. d) Precise arrangement of RBCs in the blood vessel into a pentagon‐like shape (d1), and the letters “J,” “N,” and “U” (d2–d4) for the abbreviation of “Jinan University.” e,f) Dynamic revolution and autorotation of the RBC around an external axis (e) and its own axis (f) in the blood vessel. g) Calculated optical force (*F*_o_) as a function of laser intensity (*I*) exerted on the trapped RBCs in the microfludic channel. The inset shows the calculation mechanism of the optical force through the equilibrium with fluid drag force (*F*_d_). Scale bar: 10 µm.

A schematic illustration of the optofluidic switch is shown in **Figure** **1**a. The inflowing blood encounters three branches at the vessel intersection (Figure 1a2). Normally, the blood flow will distribute into each branch without specific selectivity, because of uniform propulsion related to blood pressure. By focusing the laser beams at the vascular entrance, multiple RBCs are trapped and enriched, and act as an optofluidic switch to close branches II and III. Concurrently, the inflowing blood will be deflected directionally and flow toward branch I (indicated by red arrows, Figure 1a). The closed vessels can be turned on by decreasing the laser power, resulting in the dynamic release of the trapped RBCs. By extending the capabilities of partial closing and controlled rotation, directional focusing, and rotary actuation of the blood microflow can be achieved, respectively. Figure 1b shows a schematic of the experimental setup constructed around the scanning optical tweezers system (see Methods for further details). An inverted microscope with a charge‐coupled device (CCD) camera was used for real‐time monitoring, image capture, and video recording. Insets I and II show the schematic illustration and optical images of adult zebrafish (see Methods for sample preparation), respectively.

Zebrafish are widely used as a model organism in cancer research and nanomedicine delivery.^[^ ^40^, ^41^ ^]^ The tail and fins of zebrafish are optically transparent, and RBCs can be clearly characterized in the blood vessels. Many transgenic zebrafish lines are also available with fluorescent cell types, and tissue can be imaged at the single‐cell level,^[^ ^42^, ^43^ ^]^ offering an excellent operational platform for the in vivo characterization of the optofluidic switch. The fish remained surrounded by fluid to supply oxygen for breathing in time. The fluid environment can also prevent potential thermal accumulation induced by the absorption of laser beams, and allows a moderate manipulation without damage to the fish. Our studies also suggest that zebrafish can be maintained for several months without physiological damage after these experiments.

### 2.2 Characterization of the Optofluidic Switch

To assess the stiffness of the optofluidic switch, we performed a variety of manipulations of RBCs in vivo. Figure 1c shows the specific orientation and dynamic shift of multiple RBCs in the capillary. Ten RBCs (labeled 1–10) were randomly distributed with an arbitrary orientation (Figure 1c1). Five cells (labeled 1–5) were then re‐orientated with their long axes parallel to the vessel wall, and shifted to contact each other end to end. The other five cells (labeled 6–10) were orientated perpendicular to the blood vessel (Figure 1c2). With more RBCs re‐orientated parallel to the vessel, the blocked capillary can be recovered (Figure S1, Supporting Information). Compared with the narrow capillary, flexible operations were further investigated for multiple RBCs in the larger veins. As indicated in Figure 1d1, five cells were trapped simultaneously and then arranged into a pentagon‐like shape. The flow velocity was 30 µm s^−1^ in the direction indicated by the red arrows. Because of the anesthetic treatment, the flow rate was lower than that under normal conditions. This can be controlled by manipulating the tricaine concentration and anesthetic time to examine zebrafish under various stages of anesthesia^[^ ^44^ ^]^ (Figure S2, Supporting Information). In addition, the flowing RBCs can be arranged into shapes resembling various letters, for example, “J,” “N,” and “U” for the abbreviation of ‘Jinan University” in the same blood vessel, as shown in Figure 1d2–d4.

Except in the case of precise arrangement, RBCs can undergo dynamic revolution and autorotation, that is, rotation around the external axis or its own axis, respectively. The revolution of an individual RBC is shown in Figure 1e, for which the blood was flowing to the right at a velocity of 50 µm s^−1^ (indicated by red arrows). Notably, it was more challenging to rotate a RBC in a narrower vessel with a diameter of only 16 µm, while the RBC was successfully rotated in anticlockwise direction with a period of 0.3 s. Multiple cells, such as for the cell pentagon in Figure 1d1, can also undergo dynamic revolution around the pentagon center (Figure S3a, Supporting Information). Autorotation of the RBC is shown in Figure 1f, with a vessel diameter and flow velocity of 30 µm and 25 µm s^−1^, respectively. The cell center was fixed to prevent its 2D translation (indicated by the cyan dot), while a circular optical potential well was set with a diameter equal to the cell. After that, the RBC started to rotate around its center in an anticlockwise direction with a rotation speed of 6 rad s^−1^. Multiple cells can rotate around their centers at the same time (Figure S3b, Supporting Information), with the direction and speed under dynamic control. For quantitative analysis, the optical forces (*F*_o_) were calculated through a dynamic equilibrium with fluid drag force (*F*_d_) on the RBC, that is, *F*_o_ = *F*_d_ = 6*πrηVK*, where *r* is the projection‐area equivalent radius, *η* is the viscous coefficient of blood, *V* is the relative shift velocity, and *K* is the dynamic shape factor (see calculation details in Supporting Information). As the depth of the blood vessel embedded under the skin of the fish was not clear, we performed a microfluidic experiment that used a more rigorous measurement method to determine the relation between the applied force and laser intensity. For this experiment, we constructed a microfluidic channel with RBCs to simulate an actual vascular environment at a controlled flow rate (Figure S4, Supporting Information). The laser spot diameter was measured to be 1.8 µm by using a CCD sensor to analyze the energy distribution of optical field. Figure 1g shows the optical forces as a function of laser intensities (*I*). The optical force increased with the laser intensity, and reached a value of 10.5 pN at an intensity of *I* = 9.43 × 10^9^ mW cm^−2^.

### 2.3 Targeted Closing and Directional Enrichment of Blood Microflow

**Figure 2:**
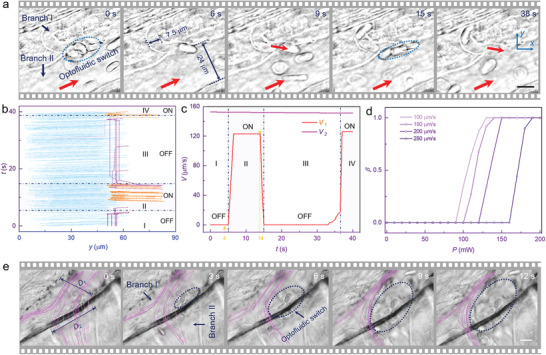
Targeted closing and directional enrichment of blood microflow. a) Assembled optofluidic switch for opening and closing branch I, with the flow directions indicated by red arrows. The diameters of branches I and II were 7.5 and 24 µm, respectively. b) Calculated *y*‐coordinate values of flowing RBCs as a function of *t*. The orange, cyan, and magenta lines indicate the RBCs flowing in branch I, II, and assembled into an “optofluidic switch,” respectively. c) Calculated flow velocity in branches I (*V*_1_) and II (*V*_2_) as a function of *t*. d) Calculated switching stability (*β*) as a function of laser power (*P*) and flow velocity (*V*). e) Directional focusing of blood microflow. The cyan lines indicate the flow line distributions in the blood vessels, with diameters *D*_1_ and *D*_2_ in branches I and II, respectively. The dashed circle indicates the assembled optofluidic switch. Scale bar: 10 µm.

The flow rate of blood was determined by changing the local blood pressure with the manipulated blood cells. Two assumption were used for the theoretical analysis of flow mechanism: i) the blood will flow from a location with high blood pressure toward that with low pressure; and ii) in fluid dynamics, an increase in the fluid speed will occur simultaneously with a decrease in static pressure (i.e., Bernoulli's principle).^[^ ^45^ ^]^ Based on these assumptions, an optofluidic switch was assembled to control blood microflow by dynamic switching of flow states (i.e., on or off). As shown in **Figure** **2**a, the two blood vessels (i.e., branches I and II) intersected each other, and several RBCs were trapped to assemble an optofluidic switch (indicated by the dashed circle). The manipulated RBCs will reduce blood flow and induce a continuous increase in blood pressure. Thus, a re‐equilibrium of pressure can be achieved for the blood in branch I, with the flow rate changed to 0 µm s^−1^ (i.e., branch I was turned off). Once the laser beam was removed, the optofluidic switch was turned on to allow blood flow from branch I into branch II, which in turn decreased blood pressure until a stable flow rate was recovered (indicated by red arrows). The laser beam was re‐introduced and removed multiple times to investigate the reproducibility of the optofluidic switch, and the blood flow was reproducibly stopped and recovered in branch I. No vessel damages or cell ruptures were detected, indicating that targeted closing of blood microflow was achievable in a noninvasive manner. The detailed operation process is shown in Video S1, Supporting Information.

Note that multiple blood flow parameters can be quantified by the characterization of RBC trajectories, including the flow distributions, directions, and magnitudes. Because blood flow was controlled at the single‐cell level, the cell trajectories can also act as biomarker for assessing hydrodynamic mechanisms and regulation flexibility. By using a high‐speed CCD camera, the experimental progress was recorded in real time (maximum frame rate: 60 Hz), and the detailed cell trajectories were calculated from video analysis using a streamline tracking method with ImageJ software. As shown in Figure 2b, the *y*‐coordinate values were investigated to discriminate the RBCs in branches I or II, with *y* > 60 µm or *y* < 60 µm, respectively. The vertical axes were set to the timeline, which provides a visualized insight into how the cell locations varied with the optofluidic manipulation. The left cyan lines, right orange lines, and vertical magenta lines indicate the cell trajectories in branch I, branch II, and “assembled into optofluidic switch,” respectively. From the cell trajectories, the flow velocities were determined for branches I and II (red and magenta lines, respectively, Figure 2c). Note that the *z* components were ignored for the calculated flow velocities because the blood vessels were not completely in plane. Nevertheless, the cells showed a distinct morphology and remained in focus, indicating only a slight fluctuation of the vessels in the *z* direction.

The dynamic configuration of the optofluidic switch was divided into four regions. First, five RBCs were trapped at the entrance (region I), and the flow velocities were *V*_1_ = 0 and *V*_2_ = 150 µm s^−1^ for branches I and II, respectively. Thus, branch I was at the “OFF” state with no RBCs flowing. After laser removal at *t* = 4 s, the trapped RBCs flowed away, with branch I switched to the “ON” state. Concurrently, RBCs began to flow out from branch I (indicated by the orange lines in region II) with an average flow velocity of 120 µm s^−1^. With the laser turned on at *t* = 14 s (region III), the RBCs were immediately trapped and remained stationary to block the blood microflow (i.e., *V*_1_ = 0 µm s^−1^). The dependence of the switching stability on laser power was also investigated. As *P* decreased from 150 to 120 mW, two trapped RBCs escaped as indicated by the magenta lines at *t* = 20 and 28 s (Figure 2b), respectively. However, the other RBCs were then trapped, and gradually added into the optofluidic switch. With further decrease in laser power, the optofluidic switch started to oscillate at *t* = 34 s, and was finally broken at *P* = 100 mW. Subsequently, blood flow was re‐established in branch I, with a velocity of *V*_1_ = 120 µm s^−1^ (region IV). In the four regions, *V*_2_ remained at 150 µm s^−1^, indicating that blood flow in branch II was independent of the optofluidic switch. Thus, a flexible closing was achieved for the targeted vessel without affecting the surrounding vessels.

A series of experiments were conducted to determine the switching stability, defined as *β* = (*N*_0_ − Δ*N*)/*N*_0_, where *N*_0_ and Δ*N* are the cell amounts assembled into and out of the optofluidic switch, respectively. Figure 2d shows the calculated *β* as a function of laser power and flow velocity. At a specific flow velocity (e.g., 150 µm s^−1^), a threshold of *P*_thr1_ = 100 mW was required to trap the RBCs and assemble them into the optofluidic switch. However, the cells could easily escape because of the flow disturbance (i.e., *β* < 1). By increasing the laser power, *β* was increased and reached 1 at *P*_thr2_ = 130 mW. *P*_thr1_ and *P*_thr2_ were also increased with the flow velocity because of the larger fluid drag force. Figure 2 shows the dynamic switching for the branch vessel (branch I) rather than the stem vessel (branch II). To demonstrate the stiffness of the optofluidic switch, the stem vessel was also investigated as it underwent a targeted closing (Figure S5, Supporting Information).

Blood flow can be enriched directionally, as indicated in Figure 2e. Two branches (I and II) intersected each other, with the blood mixing together and flowing toward the upper right region. The cyan lines indicate the flow line distribution (blood flow trajectories), which were achieved from the spatial‐temporal distribution of RBC flows. The flow line diameters were *D*_1_ = 42 µm and *D*_2_ = 30 µm for the normal blood flow at *t* = 0 s, which were consistent with those of branches I and II, respectively. The optofluidic switch was then introduced, and the vascular cross sections were partially closed (indicated by the dashed circles). Subsequently, the flow line diameters were gradually decreased, with *D*_1_ and *D*_2_ decreasing to *D*_1_ =23, 15, 8, 5 µm and *D*_2_ = 20, 14, 12, 9 µm at *t* = 3, 6, 9, 12 s. The blood flow then enriched with an increased flow velocity when passing through the narrowing vessels, suggesting a possible extension of the functional capability of the optofluidic switch with directional enrichment and acceleration.

### 2.4 Dynamic Redirecting of Blood Microflow

**Figure 3:**
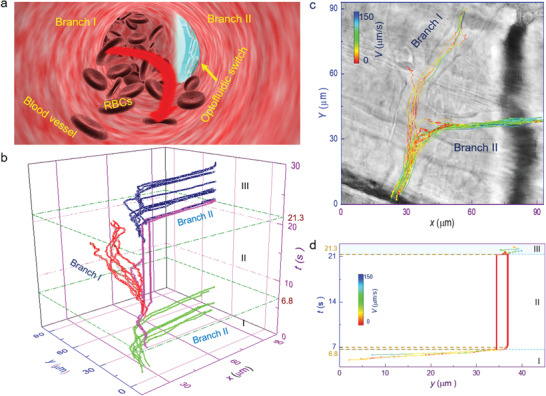
Dynamic redirecting of blood microflow. a) Schematic illustration for the dynamic redirecting of blood flow in vivo. b) Calculated cell trajectories as a function of *t*. The green, red, and purple lines indicate the RBCs flowing toward branches II, I, and II, respectively. The magenta lines indicate the RBCs assembled into the optofluidic switch. c) Superposition trajectories of RBCs in the *x*–*y* plane with the colors indicating the velocities at various locations. d) Calculated *y*‐coordinate values as a function of *t* for two trapped RBCs in the optofluidic switch.

**Figure** **3**a shows a schematic illustration of dynamic redirecting of blood flow into the targeted branches. The inflowing blood encounters two branches (I and II), and flows into them stochastically under a natural blood pressure distribution. However, branch II can be switched to the “OFF” state once the optofluidic switch is assembled at its entrance. Concurrently, blood will be “trained” to flow toward branch I in a controlled manner (indicated by the red arrow, Figure 3a). Alternatively, blood can turn to flow only in branch II, that is, a dynamic redirecting can be achieved for blood microflow. To provide a further example, a specific vessel branch was chosen to examine the redirecting mechanism, where the blood was flowing toward branch II under normal conditions. The microscope images and detailed experiment process are shown in Figure S6 and Video S2, Supporting Information, respectively. The cell trajectories were also investigated as a function of *t* (Figure 3b). The experiment was divided into three regions according to the flow direction. In region I, the blood flowed toward branch II, with flowing RBCs indicated by the green lines. At *t* = 6.8 s, the lasers were introduced to assemble one optofluidic switch at the entrance of branch II (indicated by magenta lines). The blood flow then changed direction, and the RBCs began to move toward branch I (indicated by red lines). By turning off the laser beams at *t* = 21.3 s, the trapped RBCs flowed away along the trajectories indicated by the magenta lines in region III. Concurrently, the blood flow returned to branch II again (indicated by purple lines).

A detailed superposition of the RBC trajectories in the *x*–*y* plane is shown in Figure 3c, and each cell is represented by one line. The colors indicate the cell velocity at various positions, with a maximum and minimum value of 150 and 0 µm s^−1^, respectively. Note that two routes were clearly discriminated toward branches I and II, respectively. The specific lines remained red at the entrance of branch II (i.e., a velocity of zero), indicating that the RBCs assembled into an optofluidic switch. For RBCs flowing toward branch I, the colors changed from green to orange, and then turned green again. Thus, the RBCs decelerated when they approached the optofluidic switch under the action of optical forces. We also examined the switching time *t*_s_ (i.e., the duration from laser on to vessel off) to characterize the switching stiffness. The first and last RBCs that assembled into the optofluidic switch were investigated with their *y*‐coordinate values as a function of *t* (Figure 3d). After the laser turned on at *t* = 6.8 s, the first RBC was immediately trapped, while the last one was trapped at *t* = 7 s. Thus, the optofluidic switch was rapidly assembled with *t*_s_ = 200 ms. By removing the laser at *t* = 21.3 s, branch II was immediately opened. The off‐time of branch II was *t*_off_ = 14.3 s, which can be further regulated through manipulation of the optofluidic switch.

### 2.5 Rotary Actuation of Blood Microflow

**Figure 4:**
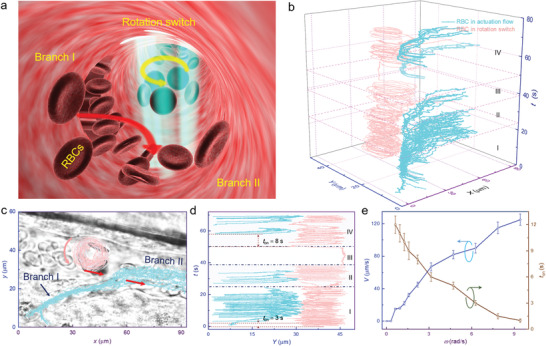
Rotary actuation of blood microflow. a) Schematic illustration of the optofluidic rotation switch for actuating blood flow in branch I. b) Calculated cell trajectories as a function of *t*. The cyan and pink lines indicate the RBCs in the actuation flow and rotation switch, respectively. c) Superposition trajectories of flowing and rotating RBCs in the *x*–*y* plane. d) Calculated *y‐*coordinate values as a function of *t*. The switching time *t*_on_ was 3 and 8 s in region I and IV, respectively. e) Calculated flow velocity (*V*) and switching time (*t*_on_) as a function of rotation speed (*ω*). Error bars represent standard deviation.

In some vascular diseases, the vessels may be blocked under a natural blood pressure equilibrium, with no blood flow.^[^ ^46^, ^47^ ^]^ However, if the RBCs were rotated at the entrances (indicated by yellow arrows, **Figure** **4**a), the local pressure will decrease according to Bernoulli's principle. We found that the blood started to flow from branch I to branch II, that is, from areas of high pressure toward areas of low pressure (indicated by red arrows, Figure 4a). By increasing the rotation speed of the RBCs, there was a larger change in blood pressure, and thus an increased blood flow rate. An optofluidic rotation switch, acting as a biological pump, was then assembled to induce the specific actuation of blood microflow, and to study the actuation mechanism in a natural blocking vessel. The microscope images and detailed experiment process are shown in Figure S7 and Video S3, Supporting Information, respectively. Figure 4b shows the calculated cell trajectories to interpret the actuation progress. The cells assembled in the rotation switch were continuously rotating, with the trajectories formed into continuous loops, as indicated by the pink lines. Meanwhile, the cyan lines indicated the trajectories of RBCs that were flowing out from branch I. When the RBCs approached the entrance, they started to flow toward the right side along branch II. The velocity of the actuation flow was dependent on the rotation speed of the optofluidic switch, as reflected by the proportional numbers of cyan and pink lines. The blood actuation was divided into four regions. In region I, the cells were rotated at a high speed, and the blood flow was rapidly actuated, with abundant RBCs flowing out from branch I. The flow velocity then started to decrease with the rotation speed in region II. The cells stopped rotating, and no RBCs were observed flowing out (region III). Finally, the rotation switch started to work again, and the blood flow returned from branch I. Thus, the blood is under a dynamic actuation, with the flow velocity controlled by the RBC rotation speed.

The superposition of the RBCs trajectories is shown in Figure 4c to interpret the flow lines in blood vessel. At the entrance of branch I, the RBCs were assembled into an optofluidic rotation switch. The rotation occurred in an anticlockwise direction, for which the tangential velocity at the entrance was consistent with the actuation flow (indicated by two red arrows). As such, the blood flow was easier to drive than rotate along the clockwise direction. The blood flow then emerged from branch I into branch II, and moved toward the right side (indicated by cyan lines). The *y*‐coordinate values of the RBCs were further investigated as a function of *t* to interpret the switching time *t*_on_ (duration from cell rotation to the emergence of actuation blood flow). As shown in Figure 4d, *t*_on_ was 3 and 8 s in regions I and IV, with a rotation speed of 2*π* and 0.7*π* rad s^−1^, respectively. Thus, a shorter duration can be expected under a faster rotation actuation. A series of experiments were then performed to interpret the dependence of flow velocity (*V*) and switching time (*t*_on_) on the rotation speed (*ω*). The velocity was increased with *ω*, and reached a peak of 125 µm s^−1^ at *ω* = 3*π* rad s^−1^ (Figure 4e). However, the switching time *t*_on_ was decreased at a larger *ω*, because of the faster decrease in blood pressure at a larger rotation speed.

## 3 Discussion

In blood vessels, dynamic blocking and clearing have been previously achieved by using the optically manipulated RBCs.^[^ ^34^ ^]^ Nevertheless, the reported strategy is restricted to the operation in capillaries and require the assistance of a vessel wall. Thus, it may be challenging to adapt to veins with diameter of tens of micrometers, because of the limited capability to manipulate multiple cell at a time. Although flexible manipulation has been demonstrated for multiple RBCs and nanoparticles,^[^ ^35^ ^]^ multifunctional control of blood flow has not been reported, especially for dynamic rotary actuation because of its inability to rotate cells in blood vessels. In the present study, we demonstrated the multifunctional micromanipulation of blood microflow by assembling an optofluidic switch using natural RBCs, which has potential for use in biomedical diagnosis and clinical therapy. With our proposed strategy, targeted blood flow can be stopped by closing the blood vessel with expected durations, providing an excellent platform to investigate endothelial functions, as well as the associated vascular development and remodeling free from the disturbance of blood flow. Furthermore, the directional enrichment can be used to enhance or prevent cell‐cell interactions between the flowing RBCs and endothelial cells of interest, providing the capacity to examine mechanisms underlying cell adhesion, cancer metastasis, and thrombus generation. In addition, dynamic redirecting of blood flow can control the motion trajectory of functionalized cells and nanoparticles in blood vessels, with potentials for the targeted drug delivery in vivo. The rotary actuation provides the basis for future studies of blood flow hemodynamics, rheology, and dynamic triggering mechanisms, which can act as a non‐contact “blood flow pacemaker.”

Because the proposed technique was applied to living organisms, we also characterized the biological safety (Figure S8, Supporting Information), and found no visual damage to the vessel or cell. This is likely because of three factors. First, the wavelength of laser beam was 1064 nm to assemble the optofluidic switch, which was within a safe window for the biological cells.^[^ ^48^ ^]^ Second, the absorption‐induced heat was effectively conducted and diffused, because of the high thermal conductance of cells and the fast blood flow in vessels;^[^ ^34^ ^]^ Third, the zebrafish remained surrounded by fluid, which can prevent local thermal accumulation because of good heat conductance and continuous fluid evaporation.

Further, we discuss the potential limitations of the proposed optofluidic switch to evaluate this technique more comprehensively. First, the manipulation depth was within ≈100 µm, above which the RBCs could not be trapped stably, and easily escaped with blood flow. Thus, it remains challenging to control the blood flow in deep tissues, because of the strengthened absorption and scattering by biological tissues. Fortunately, the technique can be further improved with the assistance of optical coherence compensation technology^[^ ^49^ ^]^ to provide a stronger focus in deeper tissues. Second, it remains difficult to manipulate blood flow in arteries of hundreds or thousands of micrometers in diameter using the proposed strategy. This is largely because the trapping sites are distributed mainly on the focal plane, that is, with limited manipulation capability in the optical axis direction. However, this can be optimized with space light modulation techniques to provide enhanced multipoint manipulation flexibility in the three directions.

## 4 Conclusion

In conclusion, we demonstrated an optofluidic switch assembled from endogenous RBCs that was applied for the multifunctional control of blood microflow in vivo. The switching characteristics, including switching time and stability, were characterized as a function of the laser power and flow velocity. Furthermore, the switch can be removed dynamically without the aggressive insertion of external devices. This label‐free, noninvasive and biocompatible switch has promise for understanding the blood microenvironment, and may provide new insight into blood diseases and development of future treatments.

## 5 Experimental Section

##### Experimental Setup

The experiment setup was constructed around a scanning optical tweezers system (Tweez250si, Aresis Co., Ltd.) integrated with an acousto‐optic deflector (AOD). A laser beam at a wavelength of 1064 nm (maximum input power: 5 W) was used to assemble the optofluidic switch in living blood vessels, because of its low absorption and weak optical damage to biological sample. The AOD was integrated to modulate the spatio‐temporal distribution of the beam focus, and the high switching rate (maximum: 100 KHz) can ensure stable trapping and precise arrangement of multiple targets at the same time. After modulation by the AOD, the laser beam was expanded by a beam expander to overfill the pupil of the microscope objective, and then reflected upward by the dichroic mirror. The laser was then refocused into the sample chamber after passing through a 60× water immersion inverted objective (CFI Apo, NA = 1.0). The experimental process was recorded using a high‐speed CCD camera with a maximum frame of 60 Hz, and displayed on a computer screen in real time.

##### Sample Preparation of Zebrafish

The Animal Experiment Protocol was reviewed and approved by Laboratory Animal Ethics Committee of Jinan University. Adult zebrafish (90 days old) were maintained under a general anesthesia by placing them in a Petri dish containing tricaine solution. The tricaine solution was prepared by diluting the tricaine in salt water at 75−300 mg L^−1^. After anesthesia for 8 min, the fish were moved to a 15 × 50 mm cover glass and then placed onto 2% low melting point agarose for immobilization. Using a pipette, ≈1 mL tricaine solution was then injected around the zebra fish to maintain a stable fluid surrounding. Excess fluid was removed from the edges using filter paper. Finally, a coverslip was carefully placed on top to allow for flexible adjustment.

##### Statistical Analysis

All data statistical analyses were performed to use the Origin software (version 2018b, OriginLab Inc., USA). The data number for each group was ≥3 and numerical data were reported as Mean ± SD.

## Conflict of Interest

The authors declare no conflict of interest.

## Supporting information

Supporting Information

Supplemental Video 1

Supplemental Video 2

Supplemental Video 3
