# Supplementary material for: In Vivo Optofluidic Switch for Controlling Blood Microflow
Source: Adv Sci (Weinh). 2020 Jun 9;7(14):2001414. doi: 10.1002/advs.202001414 (PMC7375249; doi:10.1002/advs.202001414)
Supplement: Supplementary file 1 — Supporting Information [file ADVS-7-2001414-s001.pdf]

Copyright WILEY-VCH Verlag GmbH & Co. KGaA, 69469 Weinheim, Germany, 2020.

Supporting Information

In Vivo Optofluidic Switch for Controlling Blood Microflow

Xiaoshuai Liu, Qing Gao, Yao Zhang, Yuchao Li, and Baojun Li**

1. Specific Orientation and Dynamic Shift of Multiple RBCs

Figure S1 shows the precise arrangement of multiple RBCs in a narrow capillary by using the scanning optical tweezer system. Ten RBCs, labelled 1–10, were randomly distributed in the blood vessel with a diameter of 7 μm (Figure S1a). The blood vessel was blocked because the cell diameter was larger than that of blood vessel. The laser beam was then introduced to manipulate RBCs in a controlled manner. Three RBCs, labelled 1–3, were then re-orientated with their long axes parallel to the vessel wall and shifted to contact each other end to end (Figure S1b). Further, five RBCs, labelled 1–5, were re-oriented parallel to the vessel wall. The other five cells, labelled 6–10, were orientated perpendicular to the blood vessel (Figure S1c). With more RBCs re-orientated parallel to the blood vessel, the blocked vessel was ultimately turned on, and the blood flow was recovered (indicated by red arrow in Figure S1f).

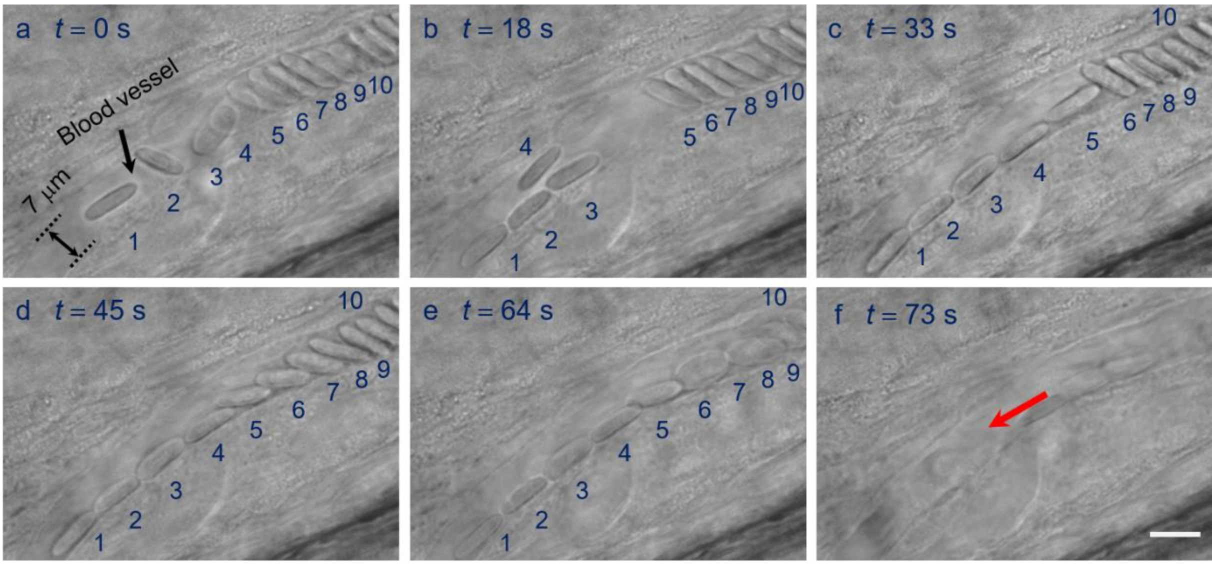

Figure S1. The specific orientation and dynamic shift of multiple RBCs in the capillary with a diameter of 7 μm. The red arrow indicated the blood flow. Scale bar: 10 μm.

2. Controlling Blood Flow Rates with Tricaine Concentration

Figure S2 shows the blood flow rate as a function of tricaine concentration with an anesthetic treatment of 5 min. All characterization were performed in a same blood vessel. The flow rate was decreased from 120 to 30 μm/s with the tricaine concentration increased from 75 to 300 mg/L. In our work, the tricaine concentration was designed to be less than 300 mg/L, because the higher concentration might cause potential mortality under long-term sedation process.

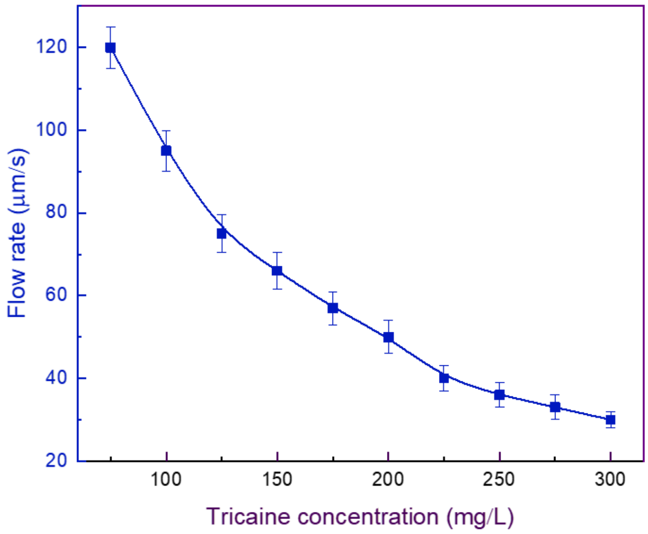

Figure S2. The blood flow rates as a function of tricaine concentration.

3. Revolution and Autorotation of Multiple RBCs

Figure S3a shows a dynamic revolution of five RBCs in the blood vessel. The RBCs were trapped one by one and then arranged into a pentagon-like shape. After that, they started to rotate around the pentagon center in a anticlockwise direction. For example, the orientation angle of RBC 1 (θ) was increased from $\theta = 50°$ to be 75°, 90°, 115° and 140° at $t = 0$ s, 0.5 s, 1 s. 1.5 s and 2 s, respectively. In addition, the autorotation of multiple RBCs was also performed in a blood capillary. As shown in Figure S3b, three manipulated RBCs were re-orientated parallel to each other, and then rotated together around their center (indicated by the blue dot). A lower velocity was achieved when the RBCs were oriented perpendicular to the vessel wall (i.e., at t = 0.5 s and 1.5 s). That was because the diameter of blood capillary was similar to the cells (i.e., 11 μm), and thus, the RBCs were hindered by the friction force exerted by the vessel wall during the autorotation.

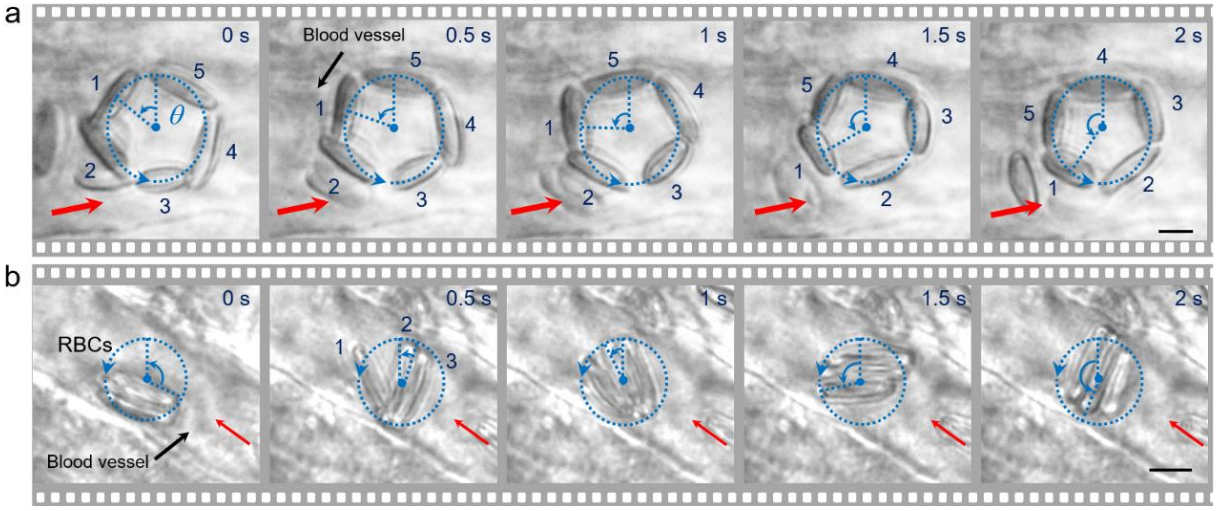

Figure S3. (a) The revolution of five RBCs around the pentagon center in a anticlockwise direction. (b) The autorotation of three RBCs around their center. The dashed circle and red arrows indicated the rotation trajectories and blood flow, respectively. Scale bar: 5 μm.

4. Calculation of Optical Force

In the blood flow, the optical force can be estimated by using the dynamic viscous drag method. Note a stable trapping of RBCs requires a dynamic equilibrium between the optical force (F_o) and fluid drag force (F_d), i.e., $F_\mathrm{o} = F_\mathrm{d}$. The fluid drag force, exerted on a spherical particle, can be directly calculated according to the Stokes' law. While for the nonspherical RBC, the Stokes' law should be extended by introducing a dynamic shape factor (K). The drag force exerted on arbitrary objects is equal to the multiplication of K with Stokes's drag for a sphere, which has the same projected area as the object in the motion direction.[1] Thus, the fluid drag force can be expressed as:

$$F_\mathrm{d} = 6\pi r \eta V K$$

where r is the projection-area equivalent radius, V is the relative shift velocity and η is the viscous coefficient of blood which is estimated to be 5×10^{-3} Pa·s at room temperature.[2]The shape factor of RBC can be expressed as: $K = 1/3\times r+2/3\times(r_s/r)$, where r_s is the diameter of sphere whose effective surface equals that of RBC.

5. Optofluidic Manipulation of RBC in a Microfluidic Channel

A schematic illustration of experimental setup is shown in **Figure S4a**. The microfluidic channel was fabricated with a glass capillary by using a heating-and-drawing method. The

inlet of microfluidic channel was connected to a microsyringe for the injection of RBC suspension, while the outlet was connected to a glass tank for the collection of effluent. The microsyringe was installed on a microfluidic pump to control the flow rates. The microfluidic channel was then fixed on a microstage under scanning optical tweezers system to perform the optofluidic manipulation of RBCs. The experiment process was displayed on a computer screen in real time with the assistance of a high-speed CCD camera. Figure S4b shows the optical microscopic image of the microfluidic channel with an inner diameters of 35 μm. The flow direction of the RBC suspension is indicated by red arrows. A RBC (marked by a blue arrow) was stationary in the microfluidics attributed by the equilibrium of optical force and fluid drag force. By adjusting the flow rate with the microfluidic pump, the correlations of the applied optical force and laser power were determined in a quantitative manner.

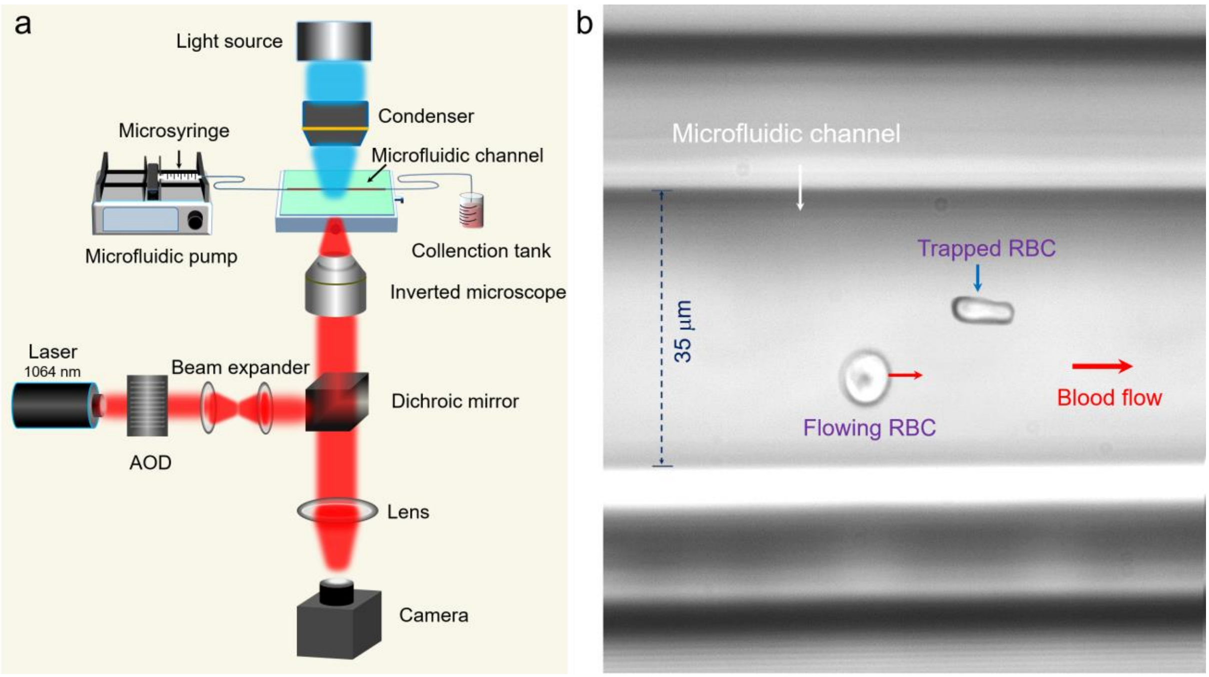

Figure S4. (a) Schematic illustration of the experimental setup. (b) Optical microscopic images of trapping one RBC in the microfluidic channel.

6. Targeted Closing of Stem Vessel

A targeted closing of stem vessel was investigated as shown in **Figure S5**. The diameters of stem vessel and branch vessel were 15 and 6 μm, respectively.The blood in branch vessel was entering into the stem vessel, and then flowing towards the right side together (as indicated by red arrows). Two RBCs were then trapped by the laser beam, and partially blocked the stem vessel (indicated by dashed circle in Fig. S5b). With five RBCs assembled into the optofluidic

switch, the stem vessel was successfully turned off at t = 8 s (Figue S5c). It then remained closed with an interval of t_{off} = 8s, and only the blood in branch vessel was flowing towards the stem vessel (indicated by red arrows in Figure S5d, e). Once the laser beam was removed, the optofluidic switch was turned on, and the blood flow was recovered in the stem vessel (Figure S5f).

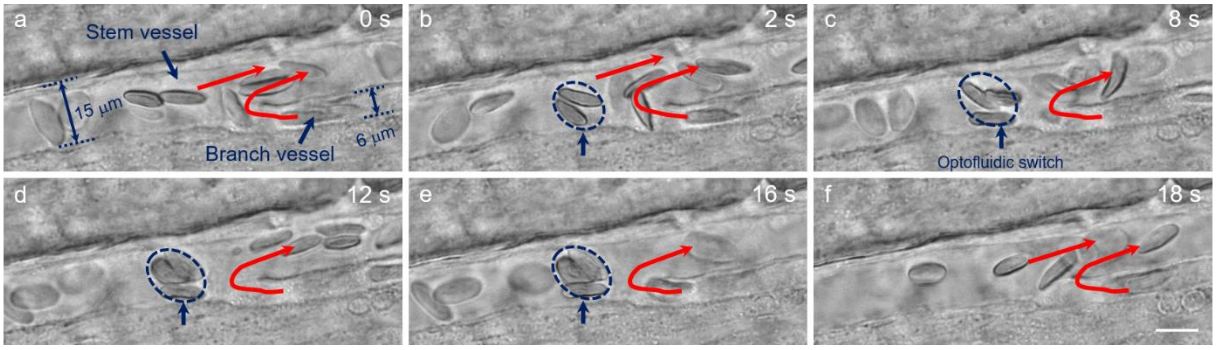

Figure S5. Optical microscopic images of the targeted closing of stem vessel. The red arrows indicated the blood flow. Scale bar: 10 μm.

7. Dynamic Redirecting of Blood Microcflow

Figure S6 shows a dynamic redirectiong of blood microflow with the assistance of optofluidic switch. At the vessel intersection, a Y-shaped route was observed and the downward blood was flowing towards the top or right, i.e., branch I or II, respectively. First, the inflowing blood was turned right, and only flowed into branch II (as indicated by red arrows). An optofludic switch was then assembled at the entrance of branch II. The blood changed its direction and started to flow towards branch I from t = 6 s to 21 s. Once the optofluidic switch removed, the blood was reflowed into branch II in a controlled manner.

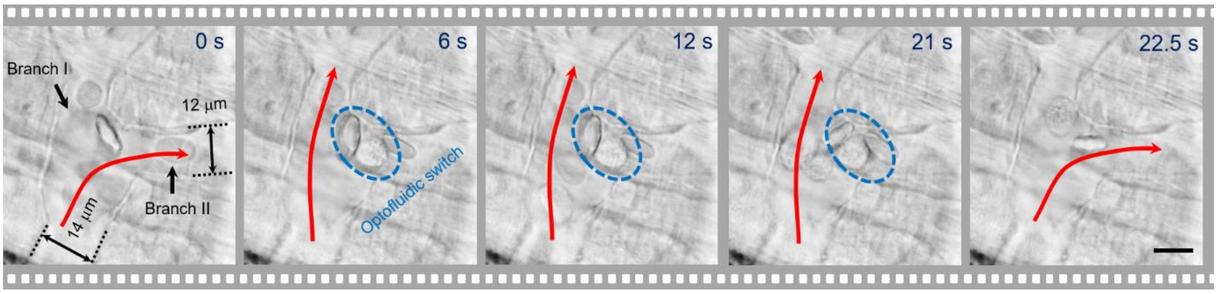

Figure S6. Optical microscopic images of the dynamic redirecting of blood microflow. The red arrows and dashed circles indicated the flow direction and optofluidic switch, respectively. Scale bar: 10 μm.

8. Rotary Actuation of Blood Microflow

Figure S7 show the rotary actuation of blood microflow in a static blood vessel. At $t = 0$ s, the RBCs remained stationary with no blood flowing in the vessel (Figure S7a). The RBCs were then trapped one by one in branch II, and started to rotate as a rotation switch (red dashed circle, Figure S7b). Meanwhile, the blood was flowing out from branch I into branch II, i.e., a rotary actuation of blood flow was achieved (Figure S7c). The flow velocity can be controlled by manipulating the rotation speed. Once the rotation switch was removed, the blood stopped flowing immediately (Figure S7d). Further, the rotation switch was re-assembled at the entrance (Figure S7e), and the blood flow was recovered (Figure S7f).

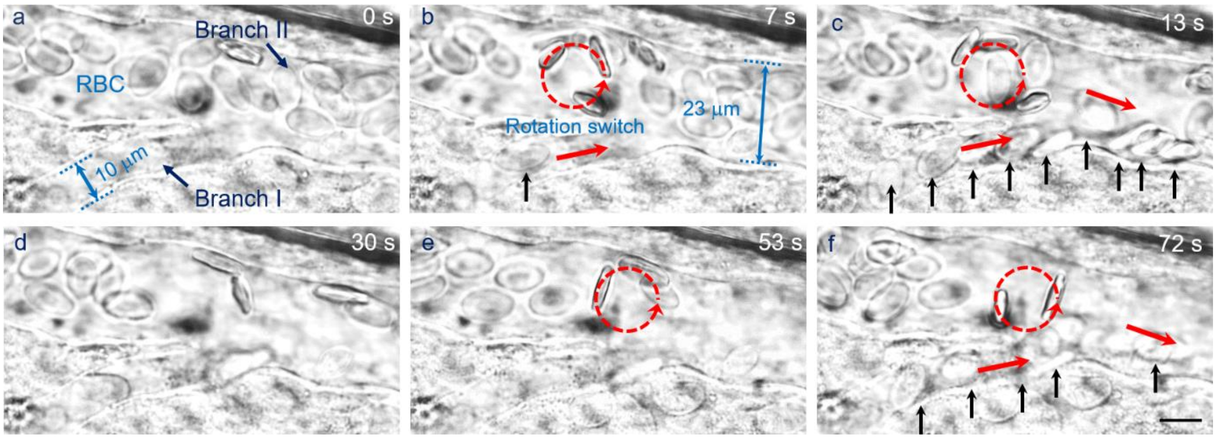

Figure S7. Optical microscopic images of the rotary actuation of blood flow. The red dashed circle and arrows indicated the rotation trajectories and flow directions, respectively. The black arrows indicated the flowing RBCs from branch I. Scale bar: 10 μm.

9. The Characterization of Biosafety

The irradiation with a high laser intensity might damage the blood vessels or RBCs. As indicated in Figure S8a, no deformation and ruptures were observed during the laser irradiation at a power of 200 mW for 20 mins, indicating there is no visual damage caused to the vessel or cell. Nevertheless, the fluid surrounding might be broken under long-term evaporation process, and then the absorption-induced heat will gradually accumlate. Figure S8b shows a visual optothermal damage to the vessel, with a bubble generation and distinct rupture under an irradiation time of 40 (Fig. S8b1) and 60 mins (Fig. S8b2), respectively. Thus, all experiments were performed within a safe range of irradiation time (i.e., 20 mins).

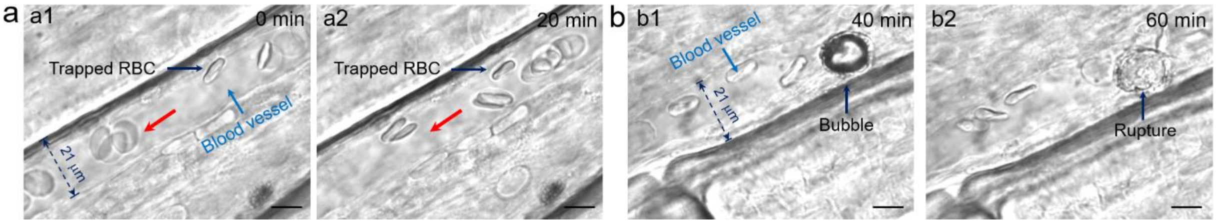

Figure S8. (a) Optical microscopic images of blood vessels and RBCs before (a1) and after (a2) optofluidic manipulation with an irradiation time of 20 min. The blood flow is indicated by the red arrow. (b) The vessels suffered from an optothermal damage with a bubble generation (b1) and vessel rupture (b2) under the irradiation time of 40 and 60 minutes, respectively. Scale bar: 10 μm.

Reference

1. Leith, D. Drag on nonspherical objects. *Aerosol sci. tech.* **6**, 153-161 (1987).
2. Chen, C.-Y *et al*. Analysis of early embryonic great-vessel microcirculation in zebrafish using high-speed confocal μPIV. *Biorheology* **48**, 305-321 (2011).
